# A *de novo* Full-Length mRNA Transcriptome Generated From Hybrid-Corrected PacBio Long-Reads Improves the Transcript Annotation and Identifies Thousands of Novel Splice Variants in Atlantic Salmon

**DOI:** 10.3389/fgene.2021.656334

**Published:** 2021-04-27

**Authors:** Sigmund Ramberg, Bjørn Høyheim, Tone-Kari Knutsdatter Østbye, Rune Andreassen

**Affiliations:** ^1^Department of Life Sciences and Health, Faculty of Health Sciences, OsloMet – Oslo Metropolitan University, Oslo, Norway; ^2^Department of Preclinical Sciences and Pathology, Faculty of Veterinary Medicine, Norwegian University of Life Sciences, Ås, Norway; ^3^Nofima (Norwegian Institute of Food, Fisheries and Aquaculture Research), Ås, Norway

**Keywords:** Atlantic salmon, transcriptome, full-length mRNA, hybrid error correction, PacBio Iso-seq, Illumina sequencing

## Abstract

Atlantic salmon (*Salmo salar*) is a major species produced in world aquaculture and an important vertebrate model organism for studying the process of rediploidization following whole genome duplication events (Ss4R, 80 mya). The current *Salmo salar* transcriptome is largely generated from genome sequence based *in silico* predictions supported by ESTs and short-read sequencing data. However, recent progress in long-read sequencing technologies now allows for full-length transcript sequencing from single RNA-molecules. This study provides a *de novo* full-length mRNA transcriptome from liver, head-kidney and gill materials. A pipeline was developed based on Iso-seq sequencing of long-reads on the PacBio platform (HQ reads) followed by error-correction of the HQ reads by short-reads from the Illumina platform. The pipeline successfully processed more than 1.5 million long-reads and more than 900 million short-reads into error-corrected HQ reads. A surprisingly high percentage (32%) represented expressed interspersed repeats, while the remaining were processed into 71 461 full-length mRNAs from 23 071 loci. Each transcript was supported by several single-molecule long-read sequences and at least three short-reads, assuring a high sequence accuracy. On average, each gene was represented by three isoforms. Comparisons to the current Atlantic salmon transcripts in the RefSeq database showed that the long-read transcriptome validated 25% of all known transcripts, while the remaining full-length transcripts were novel isoforms, but few were transcripts from novel genes. A comparison to the current genome assembly indicates that the long-read transcriptome may aid in improving transcript annotation as well as provide long-read linkage information useful for improving the genome assembly. More than 80% of transcripts were assigned GO terms and thousands of transcripts were from genes or splice-variants expressed in an organ-specific manner demonstrating that hybrid error-corrected long-read transcriptomes may be applied to study genes and splice-variants expressed in certain organs or conditions (e.g., challenge materials). In conclusion, this is the single largest contribution of full-length mRNAs in Atlantic salmon. The results will be of great value to salmon genomics research, and the pipeline outlined may be applied to generate additional *de novo* transcriptomes in Atlantic Salmon or applied for similar projects in other species.

## Introduction

Atlantic Salmon (*Salmo Salar*) is a species with significant value both economically and scientifically. It is an important aquaculture species, and there is also substantial commercial harvesting of wild salmon (FAO, 2018). Both of these activities benefit greatly from increased knowledge of salmon genetics, aiding in breeding to improve yield, quality and welfare for farmed salmon, and in monitoring the health of wild populations (Yanez et al., 2014; Abdelrahman et al., 2017; Houston and Macqueen, 2019).

Salmonids have undergone a relatively recent whole genome duplication (WGD) event, (the salmonid-specific fourth vertebrate whole genome duplication, Ss4R) approximately 80 million years ago (Allendorf and Thorgaard, 1984; Macqueen and Johnston, 2014). They are now undergoing rediploidization, which makes Atlantic salmon a model species useful for studying post WGD phenomena like rediploidization and conservation of partial tetrasomy (Lien et al., 2016; Campbell et al., 2019). Smoltification, the process by which Atlantic salmon and other anadromous salmonids adapt from life in freshwater to saltwater (Hoar, 1988), represents another scientifically interesting and unique developmental transformation that would be of interest to study by omics-technologies. This transition is also a management challenge in aquaculture due to the high mortality rate associated with the post saltwater transfer period (Hjeltnes et al., 2019). Infectious diseases caused by various pathogens are also a major challenge for the aquaculture industry and continues to lead to large economic losses and reduced fish health (Hjeltnes et al., 2019). High-quality transcriptomic resources are extremely valuable when studying the underlying molecular processes governing such developmental transformations, molecular details of infectious diseases as well as in the study of the post WGD phenomena. They are also very important resources for the continuing knowledge-based aquaculture management to improve fish welfare and ensure growth of the aquaculture industry (Abdelrahman et al., 2017).

A chromosome level assembly of the *Salmo Salar* genome has been publicly available since 2015 thanks to the efforts of the International Cooperation to Sequence the Atlantic Salmon Genome (ICSASG) (Lien et al., 2016), but the transcript-level resources are limited. There has been some work on generating full-length mRNA transcripts for *Salmo Salar* (Andreassen et al., 2009; Leong et al., 2010). The vast majority of the protein coding transcripts in the NCBI RefSeq database were, however, annotated by use of *in silico* predictions from the genome sequence supported and corrected by ESTs originating from sequencing of cDNA libraries and high-throughput sequencing (HTS) of transcriptomes on platforms producing short-read data (at time of writing, 498 177 ESTs and 4 475 852 530 short-reads) (Hagen-Larsen et al., 2005; Adzhubei et al., 2007; Koop et al., 2008; NCBI, 2015). While these methods are useful for identifying the presence of particular gene products, they are much less useful for characterizing transcript isoforms (Conesa et al., 2016). Thus, the identification of splice variants and possible misplacement of short transcript sequences on the genome due to the existence of highly similar ohnologous genes (resulting from the salmonid specific WGD) are challenges not easily solved in Salmonids if relying on short-read transcript sequencing alone (Leong et al., 2010; Liu et al., 2012). A full-length protein coding transcriptome from a species (the CDS as well as the 5′- and 3′UTRs) and its repertoire of splice variants is an essential resource to reliably annotate protein coding transcripts and understand how such structural variants impact disease and economical important traits in farmed animals (Abdelrahman et al., 2017; Giuffra et al., 2019).

Long-read sequencing based on the PacBio Iso-Seq SMRT sequencing technology produce full-length transcript sequences (e.g., full-length mRNAs) by sequencing single molecules (Rhoads and Au, 2015). This method solves the problems associated with assembly of short-read HTS data by producing reads that span the entirety of a protein coding transcript, including the CDS and the 5′- and the 3′UTRs of mRNAs. This method, thus, allows one to accurately identify different splice variants and, in salmonids, would allow one to distinguish between transcripts with highly similar ohnologous coding sequences as the complete read (including the less conserved 3′UTR) is generated from a single molecule. Being able to unambiguously characterize the 3′UTRs of specific transcript variants would also benefit the study of regulatory elements targeting these regions, such as microRNAs (Woldemariam et al., 2019, 2020; Shwe et al., 2020). The vast majority of the 3′UTRs in the present Atlantic reference transcriptome (RefSeq) (NCBI, 2015) are, however, predicted from the Atlantic salmon genome sequence with short-read support (RefSeq XM entries).

The development of the PacBio Iso-Seq SMRT sequencing technology has allowed for high-throughput long-read sequencing suitable for sequencing the complete transcriptome of a species or generate tissue specific transcriptomes to study tissue specific gene expression (Wang et al., 2016). The higher error-rates associated with long-read sequencing may be counteracted by generating a consensus out of multiple reads from a single molecule (High-Quality reads) applying the Iso-Seq method (Gordon et al., 2015; Rhoads and Au, 2015). The error rate can be further improved using graph-based hybrid error correction methods (Au et al., 2012; Salmela and Rivals, 2014; Sahraeian et al., 2017). This approach utilizes the long-reads as basis for short-read alignment that include sequence error correction. Thus, the long-reads provide the structural information of all isoforms, while the long-read isoforms are error-corrected by a much higher read number of short-reads with a superior read accuracy. This allows for the generation of a *de novo* full-length transcriptome with a quality comparable to any reference resource without the use of the current genome assembly as an error-correcting source (Feng et al., 2019). This is particularly important in non-model species where genome assemblies have, in general, considerable potential for quality improvement.

The aim of this study has been to provide the first high-quality full-length protein coding transcriptome resource for Atlantic salmon. We have a particular interest in the study of expression changes and regulation of gene expression during smoltification and sea-water transfer as well as expression changes and gene regulation in response to infectious diseases (Woldemariam et al., 2019, 2020; Shwe et al., 2020). Two of the samples included in this study were therefore selected from a challenge study to reveal the full-length sequences of mRNAs expressed in head-kidney when infected with salmonid alpha virus (SAV) (McLoughlin and Graham, 2007; Andreassen et al., 2017; Bernhardt et al., 2021). Samples from head-kidney were chosen, as this is one of the main immune organ in fish and frequently used in fish immunological studies of gene expression (Bjørgen and Koppang, 2021). The samples from the three different main stages of smoltification; pre-smolt, smoltified fish and post sea-water transfer were chosen from gills, liver and head-kidney. These are all important organs in this developmental transition, and the samples were from our recent and ongoing study of smoltification (Shwe et al., 2020). The development and evaluation of a long-read based transcriptome pipeline was another aim. We have used a combination of existing tools for sequence analysis, curation and annotation of PacBio Iso-Seq transcript data applying both sequel I and sequel II platforms. The quality of long-reads from the PacBio platform were then further improved by use of additional transcript data generated on the Illumina short-read platform. Applying hybrid error correction algorithms that were complemented with in-house developed scripts the sequence accuracy was increased. Finally, after producing a high-quality transcriptome data set that consisted of full-length mRNAs with complete CDSs, the transcripts were functionally annotated. The pipeline processing is independent of the Atlantic salmon genome sequence and other transcript sources like RefSeq for error correction. This allowed transcripts previously predicted from the genome sequence and ESTs to be experimentally validated by a long-read *de novo* transcriptome, and new splice variants and paralogs to be reliably characterized.

## Materials and Methods

### Fish Sample Materials

Table 1 gives an overview of the samples sequenced including information about the experimental condition and their organ type. The table also gives the unique labels used for each sample in the following analysis. Two of the head-kidney samples included in the study (SAV_Control and SAV_challenge, Table 1) were from one healthy control fish and one fish challenged with Salmonid Alphavirus, respectively. The challenge trial was carried out at the Industrial and Aquatic Laboratory (ILAB, Bergen High Technology Centre, Bergen, Norway) in February/March of 2018 (Bernhardt et al., 2021). Post-smolt fish from the breed SF Optimal (Stofnfiskur Iceland) were challenged by cohabitation in saltwater with salmon shedders (carrier fish) injected with salmonid alphavirus subtype 3 (SAV3) from Norway (Taksdal et al., 2015). All the fish used in the challenge trial were unvaccinated allowing the study of the immune response following viral infection with SAV. All fish tested negative for SAV3, Infectious salmon anemia virus (ISAV), Infectious pancreatic necrosis virus (IPNV), Piscine myocarditis virus (PMCV), Piscine orthoreovirus (PRV), and Salmon gill poxvirus (SGPV) prior to the challenge trial confirming that the control fish were healthy fish not infected by any of the fish virus commonly seen in aquaculture industry. The average weight of the fish was 110.9 g, and in the experimental period dissolved oxygen was between 79–97%, water temperature was between 11.5–12.4°C, and salinity was between 34.1–34.5‰ across the tanks. Samples (challenged fish and control) were collected at day 37 in the SAV challenge trial and fixed in RNA later (Life Technologies, Carlsbad, CA, United States) immediately after collection. A successful SAV3 infection was confirmed by detection of the viral sequence in the challenge sample. The experimental study was approved by the National Research Authority in Norway (NARA). All salmon used for sampling in the experiment were euthanized according to standard protocols approved by the Norwegian Food Safety Authorities prior to sampling. For simplicity, these two samples are referred to as the SAV samples.

**TABLE 1:** Distribution of tissue types and experimental conditions of samples sequenced with sample labels as used in the final dataset.

Tissue type	Pre-Smoltification^1^	Post-Smoltification^1^	Post-Seawater Transfer^1^	Control Adult^2^	SAV-challenged Adult^2^
Gill	GiU1	GiU4	GiU7	−	−
Liver	LiU1	LiU4	LiU7	−	−
Head-Kidney	HKU1	HKU4	HKU7	SAV_Control	SAV_Challenge

*The smoltification samples^1^ are from a study of Shwe et al. (2020) while the challenge samples^2^ are from Bernhardt et al. (2021).*

The nine remaining samples included were collected from fish used in a study of miRNA gene expression changes during smoltification and early saltwater period (Shwe et al., 2020). Samples were taken from the head-kidney, gills and liver prior to smoltification (day 0; HKU1, GiU1, LiU1), at the end of the smoltification (day 81; HKU4, GiU4, LiU4), and 4 weeks after sea water transfer (day 111; HKU7, GiU7, LiU7). The liver and gill samples from day 0 were from one fish while the head-kidney sample were from another. The three samples from day 81 were from same fish and this was also the case for the three samples from day 111. Details about smoltification conditions and the sample collection is given in Shwe et al. (2020). Shortly, fish were anesthetized by an overdose of MS-222 (tricaine 123 methanesulfonate, 0.1 g/L) prior to sampling and killed by a blow to the head. The tissue samples were immediately collected, frozen in liquid hydrogen and stored at −80°C. All fish handling procedures complied with the guidelines of the EU-legislation (2010/63/EU), as well as with the Norwegian legislation. The experiment was considered as a non-regulated procedure according to the National Legislation on Animal Research since the fish had not been exposed to any pain or distress. Thus, this experiment did not require application for approval from the Norwegian Food Safety Authority. For simplicity, these samples are referred to as the smoltification samples.

### PacBio Library Preparation and Iso-Seq Sequencing, Illumina Library Preparation and RNA Sequencing

The nine smoltification samples were all processed at the Earlham Institute (Norwich, England). RNA extraction was performed using the Qiagen RNeasy Mini Kit (Qiagen, Hilden, Germany), with On-column DNase Digestion using the RNAse-Free DNase Set, according to the manufacturer’s protocol. The total RNA extracts were used for both PacBio long-read sequencing and Illumina short paired-end sequencing. The PacBio non-size selected Iso-seq library preparation was used for the for long-read sequencing. The Express Template Prep 2 protocol requiring RIN-values > 8 on the RNA-samples was applied, and each of the nine samples were individually processed. The resulting cDNA-adapter complexes from the nine samples were sequenced on one PacBio Sequel II 8M SMRT Cell each. For short-read sequencing on the Illumina platform, the automated NEBNext Ultra II Directional RNA-Seq library kit with Poly-A selection (New England Biolabs, Inc., Ipswich, MA, United States) was used for library preparation, and the paired-end sequencing (150 bp) was performed using one Illumina NovaSeq 6000 SP flow cell for all nine samples multiplexed together.

The RNA extraction, library preparation and sequencing of the two SAV samples was carried out by Genewiz Germany GmbH (Leipzig, Germany). RNA was extracted with the RNeasy Plus Mini Kit (Qiagen, Hilden, Germany) according to the manufacturer’s protocol and the samples used for sequencing had a RIN > 8. For long-read PacBio Iso-seq sequencing, the cDNA synthesis was performed using the SMRTer PCR cDNA synthesis kit (Clontech Laboratories, Inc., Mountain View, CA, United States) without size-selection, and the cDNA-adapter complex was generated using the SMRTbell template prep kit V1.0. (Pacific Biosciences of California, Inc., Menlo Park, CA, United States). Each sample was sequenced using the PacBio Sequel I. Each sample was sequenced on two 1M v2 SMRT cells to compensate for the lower read count on the Sequel I compared to the Sequel II. The short-read sequencing applied the NEBnext Ultra RNA library preparation kit (New England Biolabs, Inc., Ipswich, MA, United States) in accordance with manufacturers protocol. The paired-end (150 bp) sequencing was carried out on the Illumina HiSeq 4000 platform.

### Pipeline for Generation of a Non-redundant *de novo* Transcriptome Resource With Error-Corrected High-Quality Long-Reads Free of Interspersed Repeats

**FIGURE 1:**
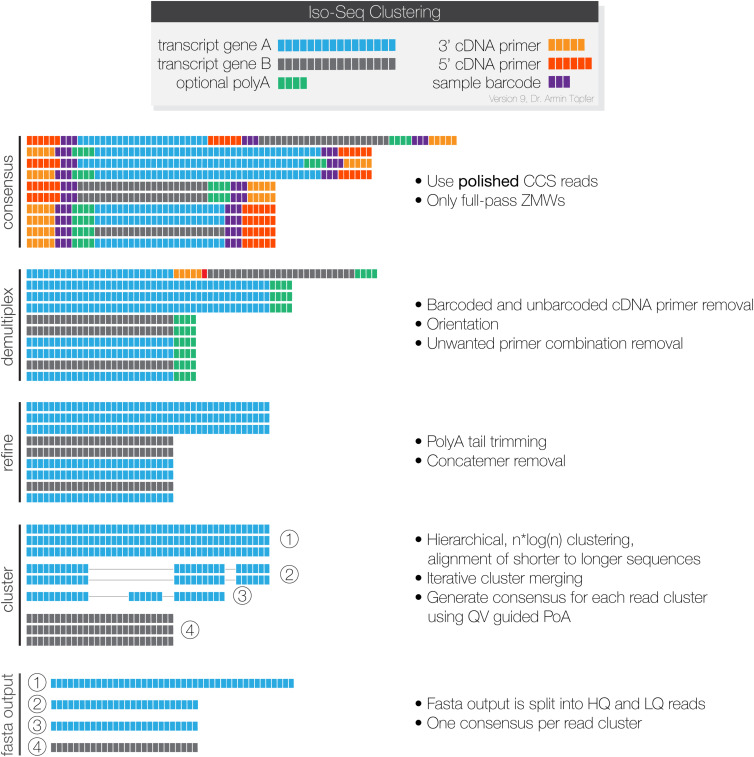
The PacBio Isoseq3 pipeline for processing SMRT-sequencing data. Each Zero-Mode Wave (ZMW) provides information from a single DNA polymerase, which sequences each cDNA-SMRTBell adapter repeatedly. Consensus: the CCS program generates a consensus sequence for each read that contains a complete repeated insert-adapter complex. Demulitplex: lima filters away sequences with unwanted primer combinations, trims away the adapter sequences, and orients the reads in the 5′→3′orientation. Refine: the refine program filters away concatemers, and sequences without polyA tails of at least 20 bp. Finally, it trims the polyA tails from the remaining sequences. Cluster: Isoseq cluster performs conservative clustering of sequences and uses partial order alignment to generate a consensus sequence for each cluster. The output is classified as High Quality or Low Quality based on the predicted accuracy. The final outputs are high quality and low-quality sequences in fastq format. This figure is used with permission from Pacific Biosciences.

The Iso-Seq raw long-reads from the PacBio Iso-seq sequencing were processed through the IsoSeq3 pipeline (PacBio, 2020) as illustrated in Figure 1. SMRT link version 8.0 was used for the smoltification samples, while 6.0 was used for the SAV samples, as they were sequenced and processed before 8.0 was released. Data from each sample was processed independently. Only High Quality (HQ) reads, meaning they were supported by at least two FLNCs and with a predicted sequence accuracy ≥ 99% (>Q20) (fasta output, Figure 1) were used in our downstream analysis.

**FIGURE 2:**
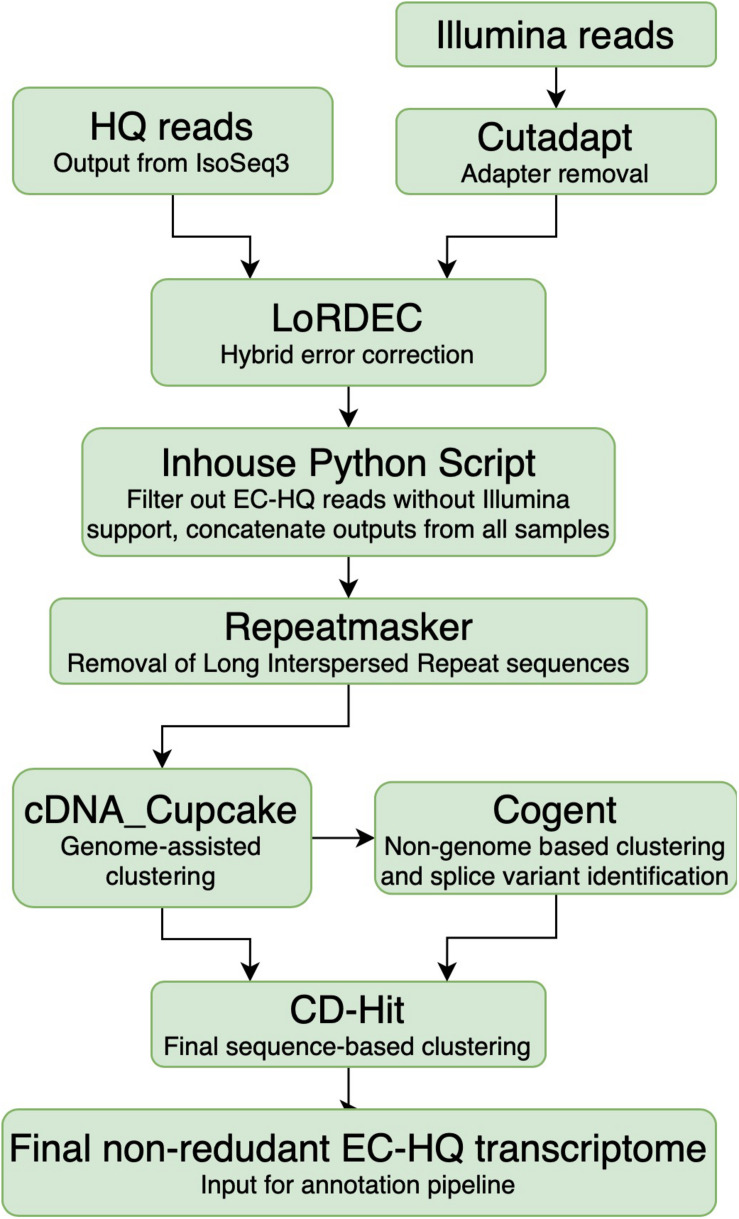
Overview of the analysis pipeline from processing of sequences up to a non-rendundant Error Corrected High Quality transcriptome. The PacBio SMRT High Quality reads were the input from the PacBio platform. The Illumina reads were first trimmed using cutadapt to remove the adapter sequences. Subsequently, they were used to generate a De Bruijn graph for LoRDEC to error-correct of the High Quality reads on a sample-by-sample basis. Inhouse python script: The error-corrected reads were filtered based on degree of Illumina support and coverage of the High Quality reads. Repeatmasker was used to identify and remove reads containing known Long Interspersed Repeats. Sequences that could be mapped accurately to the *Salmo salar* or *Salmo trutta* genome were clustered using cdna_Cupcake, while the remaining sequences were instead clustered using Cogent. All the reads were additionally clustered using CD-Hit prior to annotation. The final output was a non-redundant Error Corrected High Quality transcriptome.

Cutadapt 1.18 (Martin, 2011) was applied for adapter removal of Illumina reads and quality was checked with FASTQC (Andrews, 2010; Figure 2, Cutadapt). The high quality paired-end reads were applied for hybrid error correction of the Iso-Seq generated HQ reads using version 0.9 of the LoRDEC algorithm [Long-read De Bruijn Graph (DBG) Error Correction] (Salmela and Rivals, 2014) with the Illumina reads originating from the same sample, using k-mer size 21 and solidity threshold 3 (Figure 2, LoRDEC).

The error-corrected HQ reads (EC-HQ reads) were then filtered using an inhouse python script which removed any EC-HQ read that was less than 99% covered by the De Brujin graph generated from the Illumina reads (Inhouse python script, Figure 2). Internal sequence gaps not supported by the graph were not allowed, while one percent or less of the terminal ends were allowed to be without graph support with solidity threshold 3 (a strict filtering with 100% coverage would have demanded at least three Illumina reads to start or end at the very 5′ or 3′ terminal bases in the HQ reads). The eleven sample files with the filtered EC-HQ reads were concatenated, and each EC-HQ read was given a new unique ID number. At the same time, the sample origin and number of supporting FLNC sequences from IsoSeq3 for each new ID was noted in a separate file to make their sample origin and FLNC support traceable (Figure 2, Inhouse Python script). In practice, the error correction ensured that any base pair in the long-reads were supported by at least three Illumina reads (average phred quality 36) that consecutively covered the long HQ reads. In cases where there were differences in single base positions or small numerical differences in homopolymer stretches between the HQ read and the supporting sections of the De Brujin graph, the HQ reads were in effect corrected by the Illumina sequences.

All concatenated EC-HQ reads were searched against version 3.0 of the Dfam database (Hubley et al., 2016) using version 4.0.9 of Repeatmasker (Smit et al., 2013) as part of the OmicsBox software package, using the RMBlast search engine with default speed and sensitivity settings (Smith-Waterman score threshold of 225). Any EC-HQ read that matched any of the interspersed repeats present in bony fishes (*Actinopterygii*) were removed using an inhouse python script (Repeatmasker, Figure 2).

A grouping of the EC-HQ reads likely to originate from same genome locus was carried out applying cdna_Cupcake version 12.1.0 (Tseng, 2020a) with minimap2 version 2.17 (Li and Birol, 2018). The EC-HQ reads were aligned against the ICSASG_v2 assembly of the Atlantic Salmon genome (RefSeq accession no GCF_000233375.1) with default parameters, apart from allowing up to 10 000 bp overhangs in the 5′ end and 1000 bp overhangs in the 3′ end. This allowed any EC-HQ reads to be grouped by genome co-ordinates and assigned into groups of transcripts originating from same locus. Furthermore, reads with the same splice-pattern, representing the same isoform, were clustered together keeping the longest one as the representative isoform sequence of the cluster. The groupings of clusters and genome mapping co-ordinates were retained in the fasta headers as information that was utilized by downstream applications (SQANTI3). The relaxed 5′ and 3′ overhang cutoffs were used to allow shorter EC-HQ reads that represented fragments of other full-length EC-HQ read transcripts to be clustered together with the full-length transcripts representing this isoform, rather than being erroneously identified as separate isoform variants.

The EC-HQ reads which did not map well to the Atlantic Salmon genome were mapped against the RefSeq version of the fSalTru1.1 assembly of the *Salmo trutta* genome (RefSeq accession no GCF_901001165.1) applying cdna_Cupcake and same parameters. The remaining sequences that did not map to either the *Salmo salar* or the *Salmo trutta* genomes were mapped against the SAV genome (GenBank accession no. KC122923) as one of the individual samples was SAV infected, and the matching reads were discarded.

The remaining EC-HQ reads that did not map to either of the Salmonid genomes were clustered using Cogent 6.1.0 using default parameters (Tseng, 2020b) (Cogent, Figure 2). Cogent generates a pseudo-genome by attempting to recreate a genome sequence that could give rise to all observed transcripts in the dataset, and then clusters and groups the EC-HQ reads into families using cdna_Cupcake. In this manner, the EC-HQ reads that did not map well to either of the two genome sequences were grouped into transcripts likely to be structural variants from same gene.

All the EC-HQ reads, both the ones that were mapped to either of the genome assembly sequences as well as those grouped by Cogent were finally clustered with CD-Hit version 4.8.1 (Li and Godzik, 2006; Fu et al., 2012) (CD-Hit, Figure 2). This final clustering aligned any shorter EC-HQ read to a longer identical isoform if present in the dataset. The settings used in CD-hit alignment were:

1. Sequence identity threshold 0.99.

2. Local sequence alignment.

3. Cluster reads to most similar longer EC-HQ read if there were more than one fitting the alignment criteria.

4. The short EC-HQ read must align with 99% of its length to the longer (1% “uncovered” bases). If the shorter EC-HQ read were larger than 3000 bp the 1% limit was replaced by 30 bp or less uncovered bases.

5. The one long EC-HQ read that other shorter reads were aligned to was allowed to have any amount of overhang.

This final clustering assured that identical structural variants were aligned into a single representative EC-HQ read. In most cases the ones aligned to a longer EC-HQ reads would be 5′ incomplete EC-HQ reads or EC-HQ reads with an incomplete 3′UTR due to mispriming in the cDNA synthesis. This assured that every different structural isoform was represented by a single full-length EC-HQ read. An inhouse python script was used to identify FLNC support and contributing samples for each non-redundant EC-HQ read following the final clustering. This non-redundant EC-HQ read transcriptome was further analyzed by classification of structural variants (Materials and Methods, “Classification of Structural Variants”) and functional annotation (Materials and Methods, “Functional Annotation”).

### Classification of Structural Variants

**FIGURE 3:**
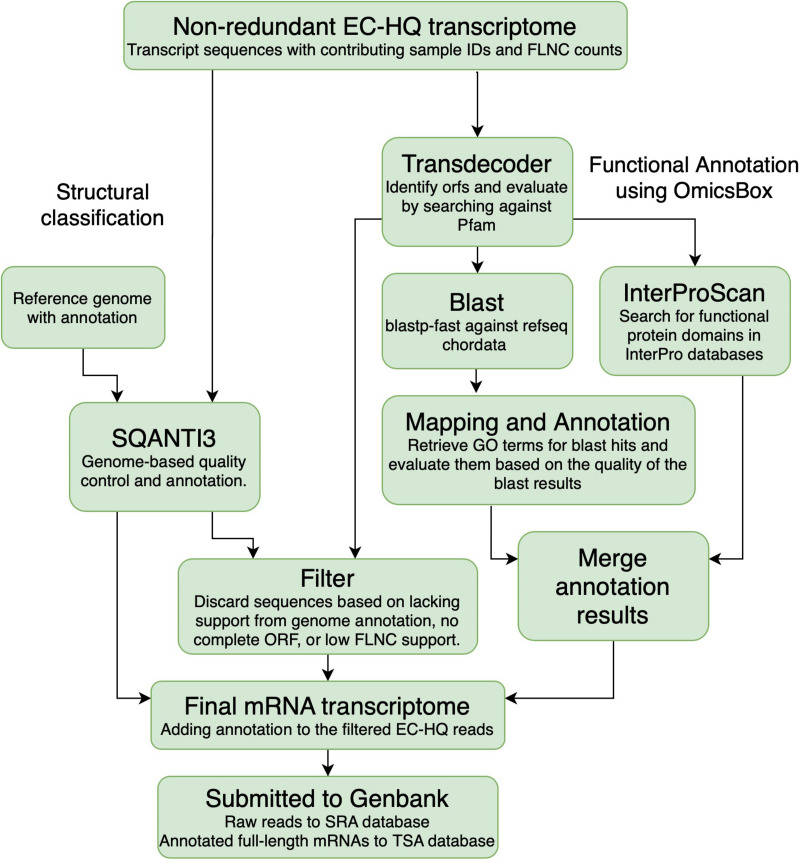
Overview of the annotation process. Transcripts that were clustered using the *Salmo salar* or *Salmo trutta* genomes were characterized against the genome annotation for the corresponding species using SQANTI3. All the sequences were also used for open reading frame prediction using Transdecoder. The sequences predicted to contain a complete coding sequence were Blasted against the RefSeq protein database and searched through the Interpro database to retrieve gene names and functional annotation. The reads were filtered based on the SQANTI-classification, open reading frame prediction and support in the PacBio sequencing data. Information from the structural classification path and the functional annotation path was added to the final filtered mRNA transcriptome.

The EC-HQ reads that had been clustered by cDNA_cupcake using the *Salmo salar* or the *Salmo trutta* genome were classified and compared against the existing annotation for the respective genomes. This analysis was carried out with SQANTI3 (v 1.0.0) with default parameters (Tardaguila et al., 2018) (SQANTI3, Figure 3). SQANTI3 compares each EC-HQ read to genome annotation information on the locus where it maps. Based on how the reads match to the genome annotation the comparisons can give the following main category classification of each EC-HQ read:

Full Splice Match (FSM) which is an identical match to a transcript isoform present in the genome annotation with all the same splice junctions and exons.

Incomplete Splice Match (ISM) representing an incomplete but otherwise identical match to a known isoform. All the present splice junctions match, but there are exons missing in either or both ends of the EC-HQ transcript.

Novel In Catalog (NIC), a novel isoform with another combination of exons than those isoforms annotated in the genome, but with a combination of known splice-junctions from previously annotated isoforms.

Novel Not in Catalog (NNC), novel isoform containing at least one splice junction not present in the annotation. Consequently, these have at least one novel exon.

Intergenic, meaning a novel transcript that maps to a locus with no previously annotated genes in the current version of the Atlantic salmon genome.

Genic intron, meaning the transcript maps entirely within the intron of an annotated gene in the Atlantic salmon genome.

Genic genomic, which means the transcript overlaps annotated introns and exons.

Antisense, which means there is no annotated gene at the locus on the strand where the transcript matches, but there is one on the reverse strand.

Fusion, meaning the read spans across two different annotated loci in the current genome annotation.

Sequences were not mapped against the *Salmo trutta* genome if they were successfully mapped against the *Salmo salar* genome. A structural classification indicating the EC-HQ was of any other category than FSM in *Salmo salar* does therefore not exclude that it would have mapped different (e.g., FSM) in *Salmo trutta.*

SQANTI also provides additional useful information like number of exons, splice junction signals (canonical or not), CDS length, polyA-signals upstream on 3′end and genomic percentage of A’s downstream of a 3′ termination site (used to judge whether there was cDNA synthesis mispriming).

### Functional Annotation

All EC-HQ reads in the final non-redundant transcriptome dataset (Figure 2) were subjected to functional annotation using the OmicsBox software suite (Figure 3; BioBam, 2019). Coding sequences were predicted with TransDecoder v5.5.0. (Haas et al., 2013; Haas and Papanicolaou, 2015) using homology search against Pfam 32 to confirm ORFs with a minimum length of 150 bp (termed complete CDS in the following manuscript if both the start and stop codon were present in-frame in the CDS). The search for ORFs was set to be strand specific, and the single best hit was kept for further analysis.

All complete CDS sequences were fed into the functional annotation workflow of OmicsBox (equivalent to the older Blast2GO software) (Gotz et al., 2008) with the following modified blast parameters: plastp-fast, species filter 89593 Craniata < chordates >, HSP-Hit coverage 70%. Default e-value cutoff for the blastp step was 1.0E-3. The HSP-Hit coverage criteria ensured that any hits were similar across the majority of the sequence, rather than just containing a highly similar partial sequence. Blast results were fed into the GO mapping and GO annotation modules. The complete CDS were also searched for functional motifs using InterProscan. The results were merged into a final functional annotation file. This process allowed us to identify mRNA transcript isoforms with complete coding regions, identify genecodes where these could not be provided by SQANTI, and provide functional descriptions of the proteins in the GO framework as well as enzyme codes.

Sequences that were mapped onto the *Salmo salar* mitochondrial sequence by SQANTI were also fed to TransDecoder using the Vertebrate Mitochodrial genetic code to search for ORFs. Any complete CDS from these sequences were also functionally annotated as described above.

Additionally, for the transcripts in the final full-length mRNA transcriptome (see section “Final mRNA Filtering Based on Supporting Evidence, and TSA Submission”), we identified subgroups of full-length mRNAs expressed in at least three samples from a specific organ type, but not in any of the other organ types in our materials. For these, we generated multi-level GO charts, showing the most specific GO-terms appearing in the dataset over the default abundance cutoffs suggested by OmicsBox, in a non-redundant way. These cutoffs were respectively 144 transcripts for gills, 98 in head-kidney, and 116 in liver.

### Final mRNA Filtering Based on Supporting Evidence, and TSA Submission

An inhouse python script (Filter, Figure 3) was used to identify EC-HQ reads that represented mRNA transcripts. The inhouse script filtered all EC-HQ reads, and only EC-HQ reads predicted to contain a complete CDS by TransDecoder were kept. Furthermore, they should be classified by SQANTI as full splice match, novel in catalog, or novel not in catalog with canonical splice junctions to be included. If classified differently by SQANTI or only grouped by Cogent, a minimum support by at least 5 FLNC reads was used as threshold for including such structural isoforms in our final full-length mRNA transcriptome. A somewhat stricter FLNC-support criteria, thus, was used for isoforms with these structural classifications, as they did not have the same level of support in the existing genome annotations as the FSM, NIC, and NNCs described above (which only required the minimum 2 FLNC support needed to be classified as HQs). The script also collected all the structural and functional annotation information for the filtered transcripts into a tsv file (Supplementary File 1).

The PacBio and Illumina raw sequencing data was submitted to NCBI’s SRA database, and the final transcript sequences in our full-length mRNA transcriptome were submitted to the Transcriptome Shotgun Assembly under the accession GIYK00000000. The version described in this paper is the first version, GIYK01000000.

### Transcriptome Comparisons Applying BLAST Analysis and SQANTI3 Annotations

Blast analysis was applied to find the degree of sequence similarity between the final *de novo* mRNA transcriptome and the *Salmo salar* mRNAs in the RefSeq database.

The complete set of RefSeq *Salmo salar* mRNA transcripts was identified and downloaded as a full record fasta files using a filtered nucleotide search through the NCBI RefSeq database. These were searched against our full-length mRNA transcriptome using blast 2.9.0 + with e-value cutoff 1e-15, and outputfmt “6 std qcovhsp slen.” The same search settings were used in the reverse comparison with our transcriptome as query against the RefSeq sequences. An inhouse python script was used to filter the blast results. The filter classified transcripts into three categories. Matches between query and subject meeting the following criteria were categorized as identical isoforms: E-value less than 10^–50^, percentage identity ≥ 99%, and either query coverage per high-scoring segment pair >99% or alignment length ^∗^ 100/subject length > 99. This ensured that any match meeting the e-value and identity thresholds had a greater than 99% coverage of the query by the subject sequence, or a greater than 99% coverage by the subject of the query sequence. These thresholds ensured that matches categorized as identical isoforms were consecutively matching sequences originating from the same isoforms, but allowed either of them to differ in UTR length compared to the other. Matches within this category were further grouped depending on whether the RefSeq RNAs had the longer UTR (query coverage per high-scoring segment pair < 99%) or whether the matching mRNA in our full-length mRNA transcriptome had the longer UTR (alignment length ^∗^ 100/subject length < 99). The second category, named significant hits, were all matches not meeting the identical isoform criteria, but with an *E*-value of less than 10^–15^. The remaining query sequences returning E-values more than 10^–15^ were categorized as non-matching RefSeq mRNAs (or non-matching full-length mRNAs in the reverse search).

The overlap between mRNAs in our dataset and the genome-annotation based mRNAs (genome reference sequence GCF_000233375.1) would be the number of sequences classified as FSM by SQANTI3. These were retrieved from the final.tsv file (Supplementary File 1). The number of FSM’s, the total listed number of mRNAs given in the genome annotation report (NCBI, 2015) and the total number of full-length transcripts in the final mRNA dataset were used to generate the Venn diagram in section “Clustering and Grouping of Unique EC-HQ Reads Revealed That 22% of These Could Not Be Mapped to the Current Atlantic Salmon Genome Sequence.”

RefSeq mRNAs with sequences that are mismatches if compared with the correlated mRNAs (exons) in the Atlantic salmon genome assembly was identified by adding the term ‘AND “assembly gap:” [All Fields]’ to the search in the RefSeq database. These represented RefSeq RNAs that are not supported by the current genome sequence. Furthermore, the number of such RefSeq mRNAs that are supported by our full-length mRNAs was retrieved by searching their accession numbers among those categorized in the identical isoform category described above.

## Results

### Hybrid Error-Correction Increased the Sequence Accuracy and Allowed for Removal of Sequencing Artifacts

The results from the Pacbio sequencing and Illumina sequencing are summarized in Supplementary File 2. As expected one Sequel II cell generated about 4–7 times more HQ reads than two Sequel I cells, but the percentage of HQ reads generated from the CCS reads were similar. Also, the size distribution of reads from the two platforms showed very similar distribution (Supplementary File 3) indicating that the reads generated by the two platforms were of equal quality. Following HQ filtering there were a total of 2 080 166 HQ reads distributed across the 11 samples (Supplementary File 2). The number of Illumina reads in all samples applied for error correction was more than 900 million with an average phred quality of 36. This resulted in a total of 1 596 834 EC-HQ reads.

**FIGURE 4:**
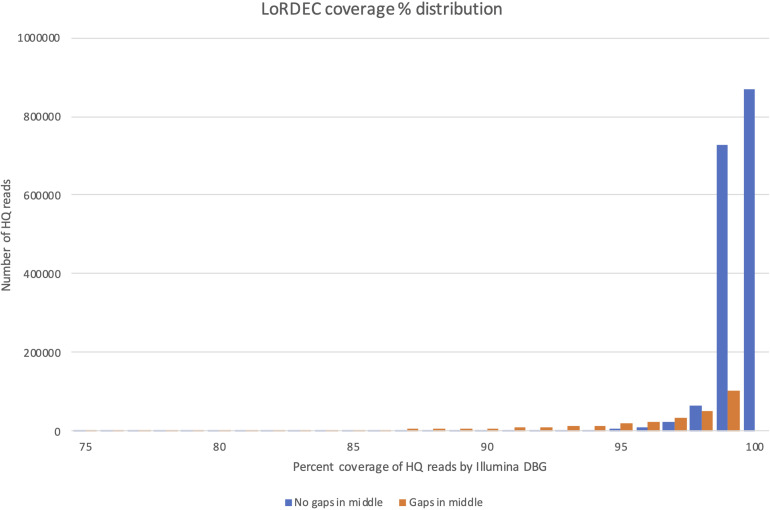
Distribution of coverage by LoRDEC for sequences with (orange bars) and without (orange bars) internal gaps in the coverage interval 75–100%.

The coverage distribution by Illumina reads on the HQ reads is illustrated in Figure 4, while the exact number of HQ reads covered by a certain percentage of Illumina reads is given in Supplementary File 4. The figure shows that the majority of HQ reads were preserved (and error-corrected) by this filtering process. More than 81% of the HQ reads had a coverage of 99% or more, illustrating that most HQ reads were error-corrected across their entire sequence. The main reason for being removed was not a general poor coverage. Instead, the sequences that were removed (23,3%) were heavily weighted toward high coverage HQ reads (95–99% coverage), but with internal small gaps in their Illumina read coverage. At 99% coverage there were, e.g., approximately 100 000 HQ reads with internal gaps not covered by the De Brujin Graph (DBG). This could indicate that these HQ reads were artifacts produced in the PacBio pipeline representing contaminating genomic sequence or fusion products (different transcripts fused together and SMRT-sequenced). This is the most likely explanation since only smaller parts of the EC-HQ sequences were not covered by the independent and much “deeper” transcriptome sequences from the Illumina platform. While the degree of single bp correction could not be directly measured we did compare the CDS lengths before and after error correction. This comparison revealed that 118 199 (7%) of the reads increased ORF size after correction, 1 455 732 (91%) had the same ORF length, and 22 903 (2%) had a shorter ORF. A substantial portion of the reads were, thus, corrected and 75% of these increased the length of their CDS. The number of short-reads applied for error correction was more than 927 million (Supplementary File 1). This is equal to 20% of all short-reads used to annotate exome sequences in the current genome assembly.

### One Third of the Transcripts Were Interspersed Repeats

The EC-HQ read dataset were analyzed by Repeatmasker to identify transcripts originating from interspersed repeats (Repeatmasker, Figure 2). After filtering out any EC-HQ read matching interspersed repeats the dataset was reduced to a total of 1 090 532 EC-HQ reads. This showed that about one third (32%) of all transcripts in Atlantic salmon are simply expressed interspersed repeats.

### Clustering and Grouping of Unique EC-HQ Reads Revealed That 22% of These Could Not Be Mapped to the Current Atlantic Salmon Genome Sequence

The mapping of the EC-HQ reads to the *Salmo salar* genome by cDNA_cupcake (Figure 2) showed an 89% success rate with 972 904 of the EC-HQ reads mapped. These were reduced to 87 315 unique reads following CD-Hit clustering. Approximately half (59 913 of 117 628) of the remaining EC-HQ reads could, however, be cDNA_cupcake clustered to the *Salmo trutta* genome. These were reduced down to 8721 unique reads by CD-Hit. The remaining 57 715 EC-HQ reads were clustered using Cogent, which were reduced to 16 367 unique reads by CD-Hit. cDNA_Cupcake and Cogent also assigned a locus number to each sequence. Thus, if they mapped to the same location on the genomes or the Cogent pseudogenome in an overlapping manner, they were grouped together as sequences that were likely isoforms of the same transcript. Altogether, the cDNA_cupcake, Cogent and final CD-Hit clustering reduced the complete dataset to 112 404 unique EC-HQ transcripts (Final non-redundant EC-HQ transcriptome, Figure 2). In summary, 78% of the unique EC-HQ transcripts were mapped to the *Salmo salar* genome while approximately 8% was mapped to the *Salmo trutta*. The remaining 14% unique EC-HQ reads (clustered by Cogent) could not be mapped to either of the two salmonid genomes. It is very unlikely that eight percent of the transcripts would map well in the Salmo trutta genome if the reason for mismatch against the Salmo salar genome was low quality or error in the EC-HQ reads. On the contrary, this indicated that there are missing or misassembled sequences in the current Atlantic salmon genome sequence that prohibited a successful mapping of a surprisingly large proportion (22%) of the of the unique Atlantic salmon EC-HQ reads. Following from this, errors in the genome sequence would be the likely explanation for why 14% of transcripts grouped by Cogent could not be mapped to the *Salmo salar* genome sequence (or the *Salmo trutta* genome). The fact that our final full-length transcriptome matches the current RefSeq mRNAs much better than the transcripts (annotated exons) in the genome sequence (identical isoforms vs. FSMs, section “Full-length Transcriptome Comparison With the Transcript Annotation of the Genome Applying SQANTI3”) also points to incorrect annotation of splice products. The *de novo* full-length transcriptome from this study may therefore aid to improve the current transcript annotation in the genome sequence. In light of this, the 22% of transcripts not currently mapped to the Atlantic salmon genome sequence represents a very useful source for long range linkage information that may be used to improve the genome sequence assembly.

### A *de novo* Transcriptome That Consisted of 71 461 Full-Length mRNAs From 23 071 Loci

The 87 315 non-redundant EC-HQ reads that had been mapped to the *Salmo salar* genome and the 8721 that had been mapped to the *Salmo trutta* genome were structurally annotated using SQANTI3 (Figure 3). The remaining sequences that had been clustered by Cogent could not be meaningfully annotated in this manner, but rather relied on the functional annotations (OmicsBox, Figure 3) for RNA category classification. Complete distribution of structural classifications for all non-redundant EC-HQ reads is shown in Table 2.

**TABLE 2:** Distribution of SQANTI-classifications and Cogent grouping for the non-redundant Error Corrected High Quality transcriptome (Figure 2) and filtered mRNA transcriptome (Figure 3).

Structural Category	*Salmo salar* Pre-filter	*Salmo salar* Final	*Salmo trutta* Pre-Filter	*Salmo trutta* Final	Cogent Pre-Filter	Cogent Final
FSM	19837	17787	1073	960	−	−
ISM	6080	2569	455	131	−	−
NIC	18338	16218	946	821	−	−
NNC	37411	22756	5443	2825	−	−
Antisense	787	256	22	8	−	−
Genic	942	385	24	5	−	−
Intergenic	2331	828	263	80	−	−
Fusion	1589	807	494	222	−	−
Cogent	−	−	−	−	16367	4803

*Sequences that could not be mapped to the Salmo salar or Salmo trutta genomes could not be classified using SQANTI. These are listed in the row Cogent. Pre-filter refers to all non-redundant EC-HQ transcripts (output from pipeline Figure 2). Final refers to the final filtered mRNA dataset (output pipeline Figure 3).*

**FIGURE 5:**
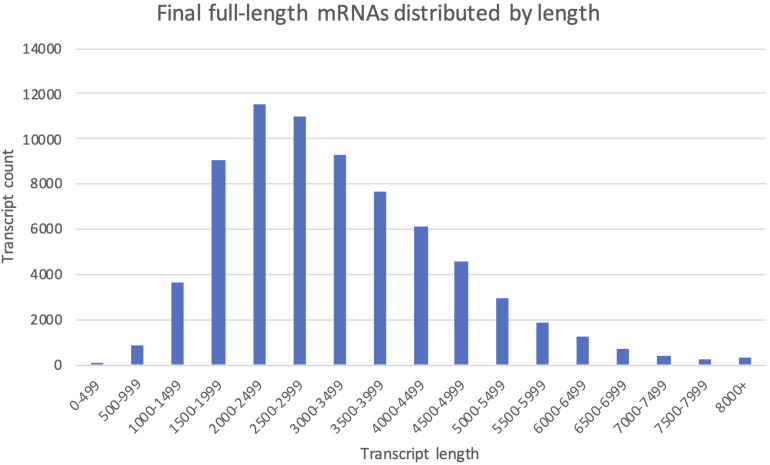
The final full-length mRNA transcriptome distributed by transcript length. Each column shows the number of transcripts falling within the given 500 bp length interval.

All 112 404 unique sequences, regardless of clustering method, were also used for ORF-prediction with Transdecoder, and further functional annotation by OmicsBox if they were predicted to have a complete CDS. Following the last filtering step (Filter, Figure 3), our final mRNA dataset consisted of 71 461 non-redundant EC-HQ reads that, by use of SQANTI3 and our OmicsBox criteria, were annotated as protein coding transcripts with a complete CDS. These transcripts were predicted to stem from a total of 23 071 loci, or likely loci in the case of Cogent transcripts, with an average of three transcripts per locus. The list of EC-HQ reads, along with SQANTI analysis outputs, sample origin, FLNC support, gene descriptions, GO codes, enzyme codes and TSA contig IDs are provided in Supplementary File 1. Despite being classified as anti-sense, genic, intergenic or fusion by SQANTI, these categories contained 2591 transcripts classified by TransDecoder as having a complete CDS and with support as full-length protein coding mRNAs by Pfam. An additional 4803 transcripts similarly classified as full-length protein coding mRNAs by TransDecoder did not map to any of the Salmonid genomes (clustered by Cogent). Sixty-seven percent of the transcripts not mapped or mapped with a non-protein coding category by SQANTI3 were also supported as protein-coding transcripts by the following GO-annotation (section “More Than 80% of the Transcripts Were Assigned GO Terms and Subsets Revealed Organ Specific Expression Patterns”) In summary, a total of 71 461 of the unique transcripts (63%) were classified as mRNAs while the remaining transcripts (37%) likely represented some other kind of non-coding RNAs. The length distribution of the mRNAs is given in Figure 5. The mRNA transcripts ranged from 319 bases to 13 331 bases in length, with a median length of 1402 bp and a mean length of 3209 bp.

The EC-HQ reads in the final mRNA dataset categorized as Full Splice Match (FSM) (final in Table 2) represented an identical match to a known isoform in the current genome annotations (*Salmo salar* or *Salmo trutta*) in terms of splice pattern and sequence identity (SQANTI3 default 95% cutoff). The majority of such matching transcripts in the current Atlantic salmon annotation have been generated from predictions based on the genome sequence with variable support from short HTS reads or ESTs (96% of *Salmo salar* RefSeq mRNAs are XM entries). The FSMs in our dataset, therefore, represents experimental validation of 17 787 transcript isoforms by single molecule sequenced full-length mRNAs. Surprisingly, there were 960 EC-HQ reads mapped as FSMs in the Salmo trutta genome. Obviously, these are also true Atlantic salmon full-length mRNAs, but inconsistencies in the current genome assembly of *Salmo salar* prevented these transcripts from mapping to the *Salmo salar* genome sequence.

The transcripts mapped as either Novel in Catalog (NIC), or Novel Not in Catalog (NNC) with exons defined by canonical splice junctions, represents novel isoforms not currently annotated in the genome sequences. The NICs have combinations of known splice sites which makes them isoforms with new combinations of annotated exons. There were 17 039 such novel transcripts in the final dataset, approximately the same number as all FSMs. There were an even larger number of NNCs, a total of 25 581 transcripts, illustrating the capability of long-read based methods to identify new isoforms not possible to predict reliably using short-reads and the genome sequence alone. Again, a substantial proportion (8,5%) of the transcripts in these categories could only map to, and be classified by SQANTI3, using the *Salmo trutta* genome.

The remaining SQANTI3 categories (ISM, antisense, genic, intergenic, and fusion) comprised the smaller part of the final mRNA transcriptome (a total of 5291 transcripts). Despite their structural classification by SQANTI3 bringing into question if these were true mRNAs, they were all supported as full-length mRNAs by OmicsBox. Also, as a threshold of at least 5 supporting FLNCs was used to filter out possible artifacts from these categories, they are likely to represent true Atlantic salmon protein-coding transcripts. Although the ISM reads were supported by the genome annotation as matching a known transcript but missing exonic sequence in the 5′ or 3′ end (or both), they all had a complete CDS. We did employ the more conservative FLNC-support criteria (at least 5 FLNCs) for ISMs, and given this threshold, it is less likely that they are incomplete, but rather represent full-length mRNAs. A small number of ISM sequences (3.7%) had 80% or higher A content in the 20 bases immediately downstream of where they mapped in the genome sequence. This could have allowed mispriming during cDNA synthesis leading to incorrect 3′UTR lengths in these transcripts. The fraction of transcripts grouped by Cogent with at least 5 supporting FLNCs is approximately as large (4803 transcripts) as those mapped to *Salmo trutta*. Again, this illustrates the ability of long-read based transcriptome sequencing to identify transcripts not detected by short-read supported genome predictions.

**FIGURE 6:**
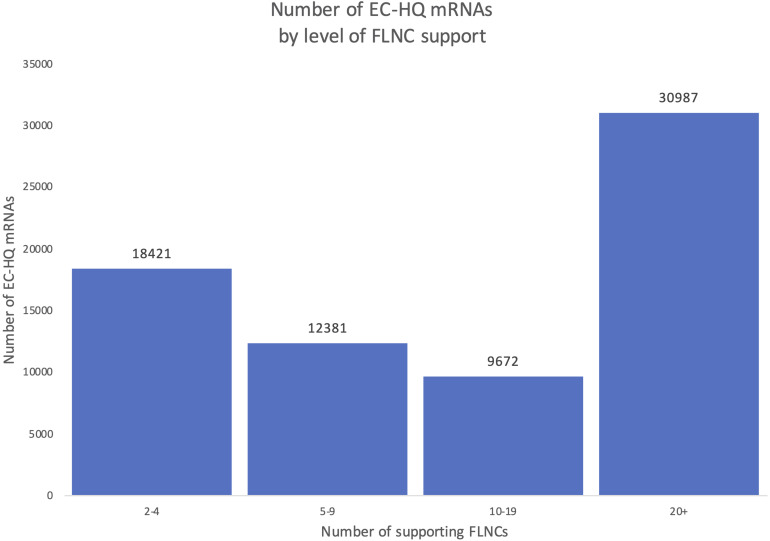
Distribution of Full-Length Non-Concatemer-support in the final mRNA dataset. Each column shows the number of transcripts in the final mRNA transcriptome with a number of Full-Length Non-Concatemer reads supporting the long-reads.

An overview of the number of FLNCs supporting each EC-HQ mRNA in the final full-length mRNA transcriptome is given in Figure 6. The figure illustrates that more than 74% had a support of more than five FLNCs independent of category. This means that approximately 70% of the FSM, NIC, and NNC categories were supported by five or more FLNCs even if the inclusion criteria for these categories was two or more FLNCs.

A python script was used to estimate the number of instances were transcripts annotated as encoding the same gene by SQANTI or OmicsBox had been assigned to different loci by cdna_Cupcake, or different gene families by Cogent. In all, 12% of genes had at least two EC-HQ mRNA transcripts that were mapped to different loci. This indicates that at least 12% of the expressed genes were likely represented by multiple paralogs in our dataset.

The transcripts have been deposited at DDBJ/EMBL/GenBank as a Transcriptome Shotgun Assembly project under the accession GIYK00000000. The version described in this paper is the first version, GIYK01000000. The TSA Contig ID for each sequence is listed in Supplementary File 1.

### Comparisons Revealed That the *de novo* Transcriptome Better Supported the RefSeq mRNA Transcripts Than the Genome Assembly Sequence

The current transcript information on Atlantic salmon mRNA sequences is provided from two NCBI sources. One is the transcript information (transcript and isoform variants defined by their exons) given in the annotation to the current *Salmo salar* reference genome sequence. The other source is the current collection of *Salmo salar* mRNA transcripts in the RefSeq database. Although these would be expected to correspond well, they differ in the sequence information given for thousands of transcripts. There are 4475 RefSeq mRNAs that are annotated as either having a gap or additional sequence that is not present in the current genome reference sequence. The long-read based *de novo* mRNA transcriptome from the present study could possibly aid to resolve what are the correct transcript sequences. The single-molecule based method applied here is also expected to be the superior one for isoform identification. This potential for increasing the quality of annotated transcript isoforms with our dataset was investigated by comparing our long-read transcriptome to each of the available sources (the RefSeq mRNA sequences and the annotation of transcripts in the RefSeq genome sequence).

#### Full-Length Transcriptome and RefSeq Transcriptome Comparisons Applying BLASTN

**FIGURE 7:**
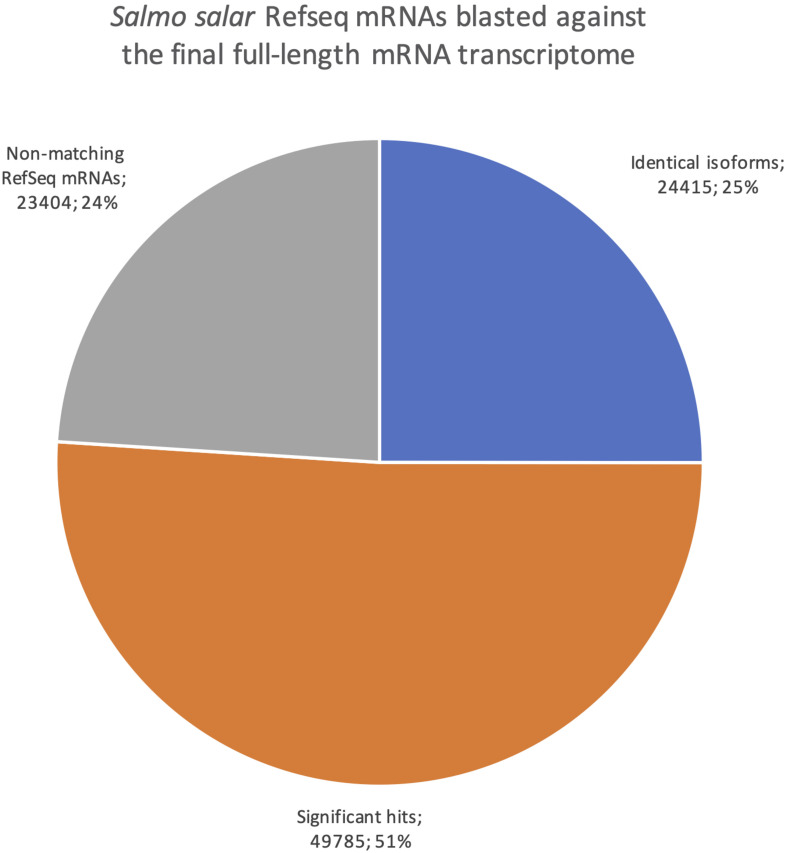
Pie chart showing the distribution of blast results when searching all *Salmo salar* RefSeq mRNAs against the final full-length mRNA dataset. Blue are identical isoforms, orange are significant hits, gray are non-matching RefSeq mRNAs.

The *Salmo salar* mRNAs in RefSeq consist mostly of transcripts that are predicted by use of the genome sequence but supported and error-corrected based on EST and short-read sequences as part of the NCBI Eukaryotic Genome Annotation pipeline^1^. All 97 604 *Salmo salar* mRNA sequences in the RefSeq database were blasted against our dataset, classifying hits into three categories (Figure 7). Identical isoforms [99% identity and coverage of the shorter matching sequence by the longer matching sequence (See section “Transcriptome Comparisons Applying BLAST Analysis and SQANTI3 Annotations” for more detail)]. The second category was significant hits, (all transcripts with e-values smaller than a 1e-15, but not meeting the very strict identical isoform criteria) and the third category was termed non-matching RefSeq mRNAs (all transcripts with e-values larger than e-15 or no hits). Filtering according to these criteria, 24 415 (25%) of the RefSeq transcripts were in the identical isoforms category, providing experimental validation for a quarter of the isoforms in the RefSeq database by full-length mRNAs from our long-read transcriptome. Furthermore, there were around twice as many mRNAs with significant hits (49 785) against our transcriptome, indicating that an additional half of all RefSeq transcripts were present as splice variants or paralogs in our dataset. Given that parts of the RefSeq mRNA sequences are predicted from the genome sequence (96% are XM entries), there is also a possibility that some of the matches in the category significant hits are, in fact, identical isoforms, but not meeting the very strict criteria we applied for this category due to sequence errors. The 23 404 mRNAs in the category of non-matching RefSeq-mRNAs likely represent transcripts from genes not expressed in the organs included in this study. The mRNAs in the category identical isoforms showed a distribution of length differences with the longer transcript being from the RefSeq dataset in 42% of cases, while the two matching transcripts deviated by less than one hundredth of their length in 28% of cases, and in the remaining 30% the longer transcripts were from our *de novo* transcriptome. These size differences were in most cases small and affected only the UTRs, not the CDSs.

**FIGURE 8:**
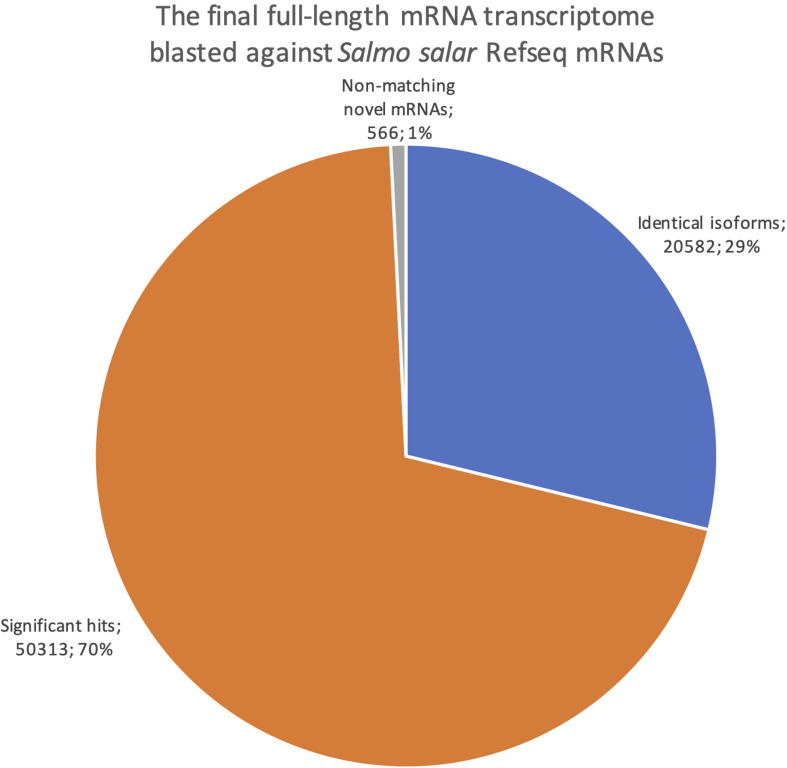
Pie chart showing the distribution of blast results when searching the final full-length mRNA dataset against all *Salmo salar* RefSeq mRNAs. Blue are identical isoforms, orange are significant hits, gray are non-matching novel mRNAs.

We also reversed the comparison, with a new blastn search where our dataset was the query sequences against the *Salmo salar* RefSeq mRNAs (Figure 8). This revealed how well our transcriptome is represented in the current RefSeq transcriptome. Notably, the number of sequences with at least one blast hit meeting the identical isoforms criteria was lower when using the full-length transcriptome as the query than when using the RefSeq mRNAs as the query (20 582, Figure 8 vs. 24 415, Figure 7). This indicates that some of our transcripts were classified as identical isoforms to multiple sequences currently listed separately in RefSeq (Figure 7). Some possible explanations for this finding are that some sequences in RefSeq are redundant, and/or that some of our transcripts have incomplete UTRs, making them unable to distinguish between some RefSeq entries. The comparison also showed that only 566 of our sequences did not have a significant blast hit (<1e-15). The result from the reversed blastn analysis, thus, indicated that our transcriptome, having 99% support in RefSeq, consisted nearly exclusively of transcript variants of known genes rather than there being transcripts from novel genes (Figure 8). Taken together, the two blast analyses showed that our full-length *de novo* transcriptome validated 25% the of currently known Atlantic salmon transcripts in RefSeq, provided a large number of new isoforms significantly matching 50% of the known transcripts in RefSeq, but did not discover many transcripts from novel genes (1%, Figure 8).

#### Full-Length Transcriptome Comparison With the Transcript Annotation of the Genome Applying SQANTI3

**FIGURE 9:**
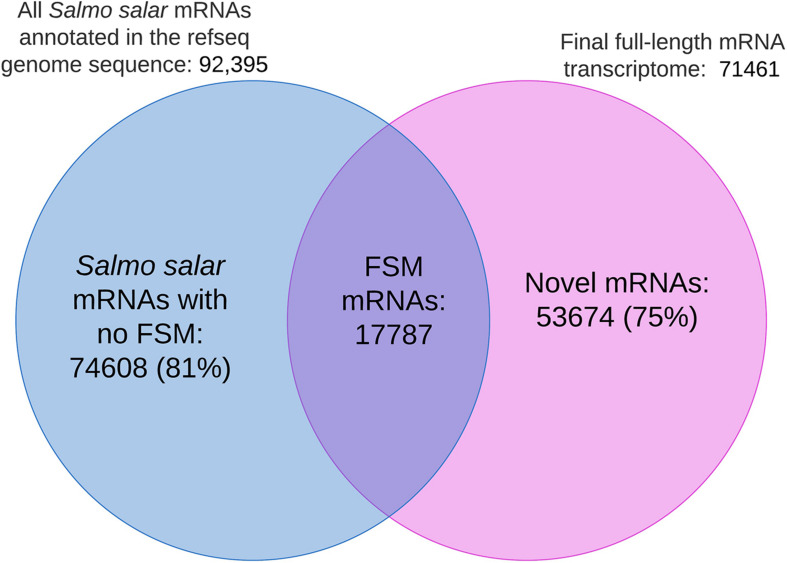
Venn diagram illustrating the number of identical isoforms (Full Slice-Match) shared among the mRNAs in the genome annotation and the final full-length mRNA transcriptome. No FSM represents isoforms in the genome annotation with no identical match in the final full-length mRNA transcriptome. Novel mRNAs refers to transcript isoforms in the final mRNA dataset without an identical match to the sequences annotated to the *Salmo Salar* genome assembly.

Figure 9 shows the distribution of shared isoform transcripts when comparing our full-length transcriptome with the mRNAs in the *Salmo salar* genome annotation. The figure illustrates that 17 782 EC-HQ mRNA transcripts were full splice matches (identical) to already annotated transcripts in the RefSeq version of the *Salmo salar* genome assembly. The majority of mRNA isoforms predicted in the *Salmo salar* genome annotation (81%, *Salmo salar* mRNAs with no FSM in Figure 9) could not be verified by our long-read mRNA transcripts. Furthermore, there were 53 674 mRNAs in our final dataset (75%, Novel mRNAs in Figure 9) that represented novel isoforms that mapped to the genome (61%, non-FSM categories in *Salmo salar* in Table 2), or mRNAs that did not map at all (14%, *Salmo trutta* or Cogent categories in Table 2) due to inconsistencies at the genome sequence level.

The fact that there were considerably fewer FSMs (19%, Figure 9) than the number of identical isoforms (25%, Figure 7) could indicate that the genome sequence is the less reliable source for transcript sequences. This is also supported by the fact that 14% of the mRNA transcripts did not map at all (mapped to *Salmo trutta* or grouped by Cogent). Furthermore, 1268 of the transcripts that did not map to the *Salmo salar* genome were in the category identical isoforms in the blast comparisons against the RefSeq mRNAs. A similar comparison where the RefSeq transcripts not matching the genome reference sequence (4475 transcripts, methods, “Transcriptome Comparisons Applying BLAST Analysis and SQANTI3 Annotations”) were compared to our *de novo* transcriptome showed that 673 of these were in the category identical isoforms. All together, these comparisons indicate that the full-length *de novo* transcriptome may contribute considerably in improving the current transcript annotation quality and aid in improving the reference genome assembly.

### More Than 80% of the Transcripts Were Assigned GO Terms and Subsets Revealed Organ Specific Expression Patterns

**FIGURE 10:**
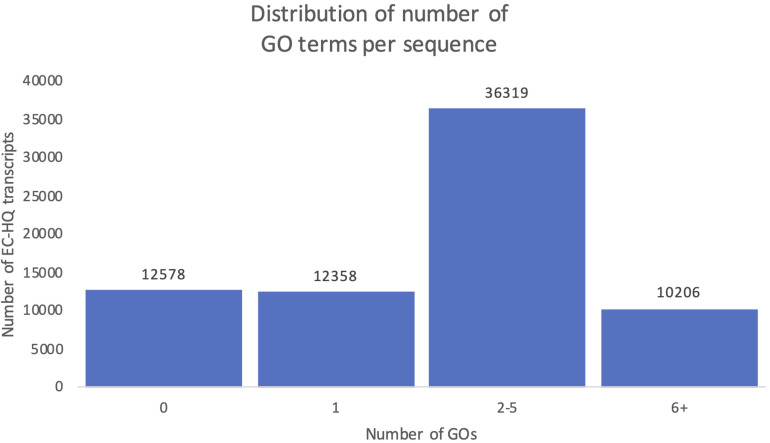
Distribution of number of predicted Gene Ontology terms. Each column shows the number of transcripts falling within the given interval of Gene Ontology terms identified for the transcript.

Figure 10 shows the distribution of OmicsBox annotation results in the final mRNA dataset. Eighty-two percent of the transcripts were successfully annotated with at least one GO term (GOs > 0, Figure 10). The remaining 18% of the transcripts with no GO terms were distributed into ones with significant blast hits, but to proteins with no GO terms in the Gene Ontology database (9%), while the other half did not have any significant hits in the RefSeq protein database. Instead, they were supported as protein-coding by their CDS length and the support in Pfam. The functional annotation, including the GO terms and gene symbols are included in Supplementary File 1. The distribution of the number of GO terms assigned to each sequence showed that 50% of the transcripts in the dataset were assigned between 2 and 5 GO terms, while 14% were assigned even more. Together there was a solid level of functional annotation for about two thirds of our dataset. A total of 46 769 of these were also annotated with specific gene symbols based on the Blastp results from OmicsBox.

Ten percent of all FLNCs in the dataset were from only 17 mRNAs (listed in Supplementary File 5). This demonstrated that there were a few highly expressed protein-coding transcripts in the final mRNA transcriptome. The single most abundant transcript, *alb1*, constituted 3,8% of all FLNCs on its own, and the top five most abundant transcripts represented 6.5% of all FLNCs. Serum albumin (*alb1*) and the two other top expressed genes (*fgg* and *itih3*) were all encoding secretory proteins from liver tissues. Other highly expressed genes like two splice variants of actin (*actb*), selenoprotein P (*SelP*), and apolipoprotein Eb (*apoeb*) were expressed in all tissues. A surprising finding was that one of the highly expressed transcripts annotated as complement factor H-like (*cfhr5*) did not map to the Atlantic salmon genome sequence, but was a FSM in the *Salmo trutta* genome. Furthermore, two other highly expressed transcripts (prothrombin-like and transferrin A-like) were annotated as fusion-products by SQANTI. Again, this is likely due to incorrect genome-annotation rather than due to sequence artifacts, given the high number of FLNCs supporting these transcripts in several tissues.

**FIGURE 11:**
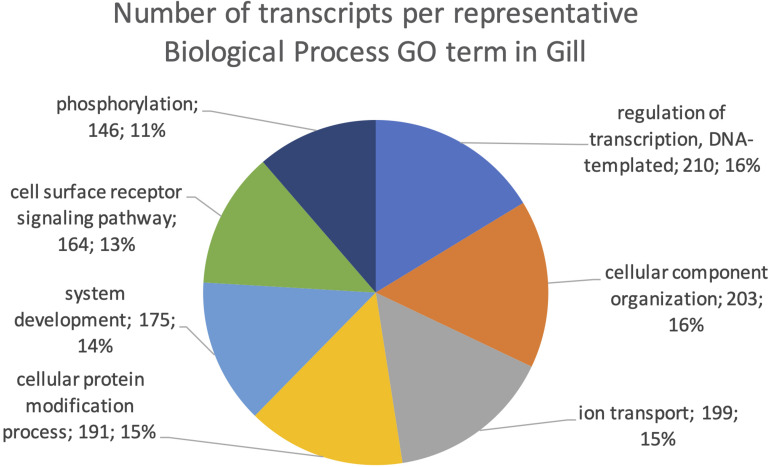
Multilevel Gene Ontology chart, gill. The pie chart shows the most specific Gene Ontology terms occurring in at least 144 gill-specific transcripts in a non-redundant way (see also Materials and Methods “Functional Annotation”).

**FIGURE 12:**
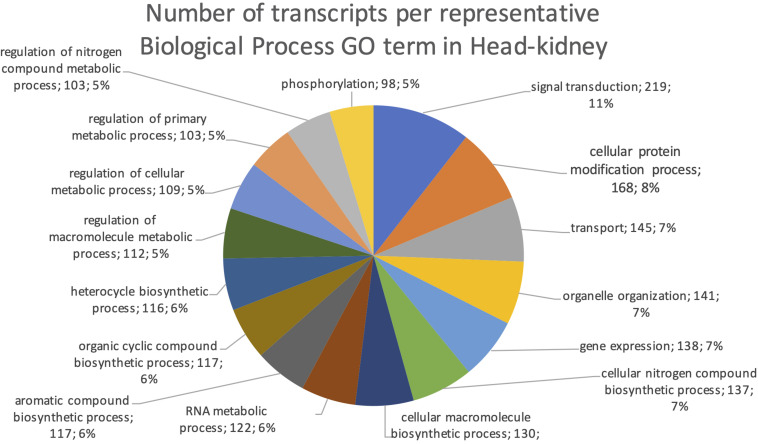
Multilevel Gene Ontology chart, head-kidney. The pie chart shows the most specific Gene Ontology terms occurring in at least 98 head-kidney-specific transcripts in a non-redundant way (see also Materials and Methods “Functional Annotation”).

**FIGURE 13:**
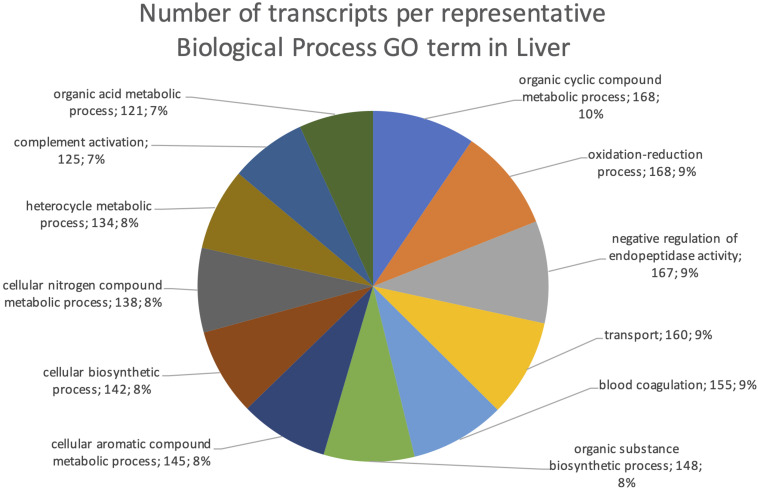
Multilevel Gene Ontology chart, liver. The pie chart shows the most specific Gene Ontology terms occurring in at least 116 gill-specific transcripts in a non-redundant way (see also Materials and Methods “Functional Annotation”).

The final mRNA transcripts were from three organs: liver, gills, and head-kidney. Several of the highly expressed transcripts were only present in liver samples, indicating that the transcriptome pipeline could identify transcripts expressed in an organ specific manner. Applying a conservative approach to identify such organ specific transcripts, we searched the full-length mRNA transcriptome for transcripts that were expressed in at least three samples from one organ, while they were absent in samples from either of the other two organs. This revealed that there were 2717 transcripts expressed in gills only, either from genes expressed only in gills (1811) or splice-variants expressed only in gills (906). In liver there were 1784 transcripts, of which 1113 were from liver-specific genes and 671 were liver-specific splice-variants. In head-kidney there were 1757 transcripts, 700 from head-kidney specific genes and 1057 were head-kidney specific splice-variants. Figures 11–13 show the distribution of the most specific common Biological Process GO terms (see Materials and Methods “Functional Annotation”) for these three organ-specific groups of mRNAs. Each GO term indicates biological processes that are enriched among the organ-specific transcripts. The GO-terms in gill transcripts clearly pointed toward gill specific functions, such as ion transport and cell surface receptor signaling pathway, genes which take part in osmoregulation. Transcripts annotated as playing a role in system development (e.g., the development of specific tissues types and regulation of DNA transcription) were also specifically expressed in the gill samples (Figure 11). The head-kidney in teleosts consists of several tissues with different distinct functions such as excretion, steroid biosynthesis, and immune response. Among the transcripts specifically expressed in head-kidney were those involved in biosynthesis of macromolecules, aromatic- and nitrogen compounds. Many were also annotated as involved in organelle organization and transport (Figure 12). There were 132 immune function and immune response genes expressed only in head-kidney, although not recognized as a uniform group of immune genes by the GO methods applied here (most specific common GO terms). Some examples of such transcripts include VIG2, different INFs, chemokines and toll-like receptors. The transcripts in liver (Figure 13), showed GO terms related to processes like metabolism, biosynthesis, and blood coagulation (e.g., the highly expressed *fgg* and *itih3*). Again, the transcripts exclusively expressed in this organ were, as expected, among those coding for proteins associated with liver function. Taken together, the results here showed the potential of our transcriptome pipeline to identify genes and splice variants that have particular organ-specific functions.

## Discussion

### The Benefits of Applying Hybrid Error Correction of Single Molecule Long-Reads in Transcriptome Sequencing

Our pipeline aimed to generate a full-length mRNA transcriptome with reference level sequence accuracy from a variety of organ samples by processing PacBio long-reads that were hybrid error-corrected with Illumina paired-end reads from the same samples. Similar approaches have been used to generate high quality transcriptomes in other species (Feng et al., 2019; Puglia et al., 2020), but this is the first of its kind in Atlantic salmon. The strategy and the main functions of the pipeline is illustrated in Figures 1–3. First, the initial generation of consensus sequences from single molecules, removal of artifacts, and conservative clustering were achieved by use of the SMRTLink package IsoSeq3 (PacBio, 2020; Figure 1). This processing corrects for much of the high raw error rate associated with PacBio sequencing [estimated to be 11–14% (Roberts et al., 2013)]. The output is sequences (termed HQ reads) that are supported by at least two single sequenced molecules with a predicted accuracy of at least 99%.

The initial examination of the HQ reads in the materials indicated there were many cases of frame shift errors leading to incorrect CDS’s or premature stop codons (data not shown). While we could not conclude for certain that these were sequencing errors in all cases, such errors were not unexpected given a sequence accuracy of 99% and the fact that PacBio reads are prone to numerical errors in homopolymers (Tedersoo et al., 2018). This accuracy would not be sufficient for our purpose, as we aimed to generate transcript sequences with a quality equally as high as (or better than) the Atlantic salmon mRNAs in RefSeq. Applying an approach where the long-reads were error-corrected using short-read sequencing data followed by filtering of sequences not supported by both datasets seemed to be the better solution (Au et al., 2012). The error correction approach takes advantage of the superior performance of the Pac Bio platform to identify structural isoforms (and differ between very similar paralogs) by long-read single molecule sequencing (Liang et al., 2016) while the accuracy is expected to be greatly increased due to a much higher read depth and phred quality contributed by the shorter reads. Also, the kind of errors most frequently acquired from the two platforms are not the same, and the probability of acquiring the same contaminating sequence from genomic DNA or other fusion artifacts when processing the same sample in two independent cDNA synthesis and different library prep methods is small. Together, transcript sequences generated from separate processing methods of sample RNA likely reduced the probability of retaining sequences with errors generated in each of the two sample processing pipelines in the final filtered EC-HQ reads (Figure 2).

A previous study (Sahraeian et al., 2017) comparing a variety of tools for RNA-seq analysis identified LoRDEC (Salmela and Rivals, 2014) as an efficient and accurate tool for hybrid error correction, and LoRDEC was successfully implemented in our pipeline. We applied a filtering of EC-HQ reads that assured that long-reads with internal gaps not supported by the shorter reads were removed. Together, the error correction approach with a very solid short-read support and the additional lower threshold for FLNC support assured that the final *de novo* transcriptome sequences had, in agreement with findings in similar studies (Au et al., 2012), an accuracy comparable with other Atlantic salmon transcript reference sources.

This pipeline focused on characterization of protein coding RNAs. The RepeatMasker software package was therefore implemented in our pipeline to ensure the final sequences would not contain transcripts from long interspersed repeats. A third of the sequences were identified as some kind of long interspersed repeat transcripts. This was a surprisingly large proportion of all transcripts. Future transcriptome projects in Atlantic salmon would benefit greatly from removing such transcripts prior to cDNA synthesis and library prep (Zhulidov, 2004). Also, after filtering with RepeatMasker, a substantial proportion (37%) of the unique EC-HQ reads were still not classified as protein coding transcripts. This revealed that our pipeline likely identified thousands of long non-coding RNAs (lncRNA). Characterization of lncRNAs by long-read approaches has emerged as the gold standard for studies of lncRNA (Wan et al., 2019), and characterization of the Atlantic salmon lncRNAs from these materials is now ongoing in a parallel project (manuscript in prep).

### The Full-Length mRNA Transcriptome Substantially Increased Number of Isoforms

Whether comparing our data with the genome sequence annotation (SQANTI3) or the blast analysis against the Atlantic salmon RefSeq mRNAs, about 70% of the final mRNA transcriptome are novel isoforms. These novel isoforms are either splice variants, (each locus had on average three splice variants) or paralogs. This showed that our long-read transcriptome sequencing approach led to a substantial increase in number of Atlantic salmon transcript isoforms. Such high success rate in discovery of novel isoforms agrees well with findings from similar studies (Zhang et al., 2019). A cutoff of 5 supporting FLNCs was implemented to filter the remaining transcript categories. Although the standard cutoff recommended by the developer is 10 FLNCs (Tseng, 2020a), other recent studies argue that 5 FLNCs is enough to support categories like fusion transcripts (Nattestad et al., 2018). We concluded that the hybrid error correction step with a minimum of three supporting Illumina reads across the entirety of the sequence provided the additional supporting evidence needed to accept the remaining SQANTI categories and the Cogent transcripts when supported by 5 FLNCs. Although these were categorized as dubious mRNA transcripts by SQANTI3 or not mapped at all, we find it likely that these are also true full-length mRNAs, but that they are not correctly annotated in the current genome sequence.

### The Long-Read Transcriptome as a Reference to Study Expression of Splice Variants and Paralogs From Organs or Particular Conditions

A large fraction of the contributing FLNCs in this study belonged to a few transcripts with extremely high levels of expression (10% of FLNC reads were from the 17 most abundant transcripts). In future projects aiming to characterize all full-length mRNAs in a sample material, removal of such transcripts (as well as all interspersed repeats) would greatly increase the likelihood of identifying the more rarely expressed transcripts (Zhulidov, 2004).

The sequencing depth applying one Sequel II cell was approximately eight times higher than from one Sequel I cell. This is in agreement with other studies (Castaño et al., 2020; Lang et al., 2020). Combining the high read depth from Sequel II with normalization methods to remove abundant transcripts one would expect most transcripts expressed in a sample to be detected. The results from this study demonstrated that our pipeline had the ability to identify a large number of transcripts expressed uniquely in each of the three organ types included in the materials. The following functional annotation also showed that the most common GO terms annotated with the transcripts were largely consistent with the function of those organs. Furthermore, any materials investigated by this long-read approach may not only identify genes expressed uniquely, but also uniquely expressed splice variants. This was also demonstrated in the group of transcripts uniquely expressed in a single organ.

Full-length transcriptomes have a range of useful applications (Oikonomopoulos et al., 2020). Among those mentioned, we propose that our high-quality full-length transcriptomes may serve as references in expression analyses. The Atlantic salmon genome sequence may be less suitable as such a reference as a very large proportion of the transcripts in this study did not align properly. Differing between splice variants or paralogs by aligning short-reads to the genome sequence would be prone to error (if not impossible). Instead, the error-corrected long-read transcriptome representing unique well-characterized transcript variants could be applied as a reference to which transcript sequences from short-read sequencing platforms, that provide greater read depth at affordable costs, could be aligned, counted and analyzed by tools like DESeq2 (Love et al., 2014). The added advantage of such analysis is that they would simultaneously detect SNP-variation. The UTRs are a rich source for such variation (Andreassen et al., 2010) and such mapping has the potential to reveal allele specific transcription, be applied to discover QTLs and even reveal causative variation leading to phenotypic differences in groups compared.

In conclusion, the hybrid corrected long-read pipeline employed here successfully generated high-quality full-length mRNA transcripts. The long-read approach led to the detection of novel splice variants and validated a quarter of all predicted Atlantic salmon mRNAs by transcripts originating from single molecule sequenced long-reads. Comprised solely of mRNAs with complete CDSs, more than 80% were assigned GO terms, and thousands of genes or splice variants from genes expressed in an organ specific manner were identifies. This full-length transcriptome will be an important resource for functional genomics in salmon aquaculture research.

## Data Availability Statement

The datasets presented in this study can be found in online repositories. The names of the repository/repositories and accession number(s) can be found in the article/Supplementary Material.

## Ethics Statement

The animal study was reviewed and approved by the National Research Authority in Norway (NARA).

## Author Contributions

SR, RA, BH, and T-KØ contributed to conceptualization and final editing of the manuscript. SR and RA contributed to analysis pipeline design, formal analysis, and wrote, reviewed and edited the manuscript. SR contributed to pipeline implementation, additional programming, and writing the original draft. RA, BH, and T-KØ contributed to resources. RA supervised and acquired funding. RA and BH contributed to project administration. All authors contributed to the article and approved the submitted version.

## Conflict of Interest

The authors declare that the research was conducted in the absence of any commercial or financial relationships that could be construed as a potential conflict of interest.

## Footnotes

1 https://www.ncbi.nlm.nih.gov/genome/annotation_euk/process/
