# Supplementary figures and images for: A de novo Full-Length mRNA Transcriptome Generated From Hybrid-Corrected PacBio Long-Reads Improves the Transcript Annotation and Identifies Thousands of Novel Splice Variants in Atlantic Salmon
Source: Front Genet. 2021 Apr 27;12:656334. doi: 10.3389/fgene.2021.656334 (PMC8110904; doi:10.3389/fgene.2021.656334)

# Length distribution of Head-Kidney PacBio reads

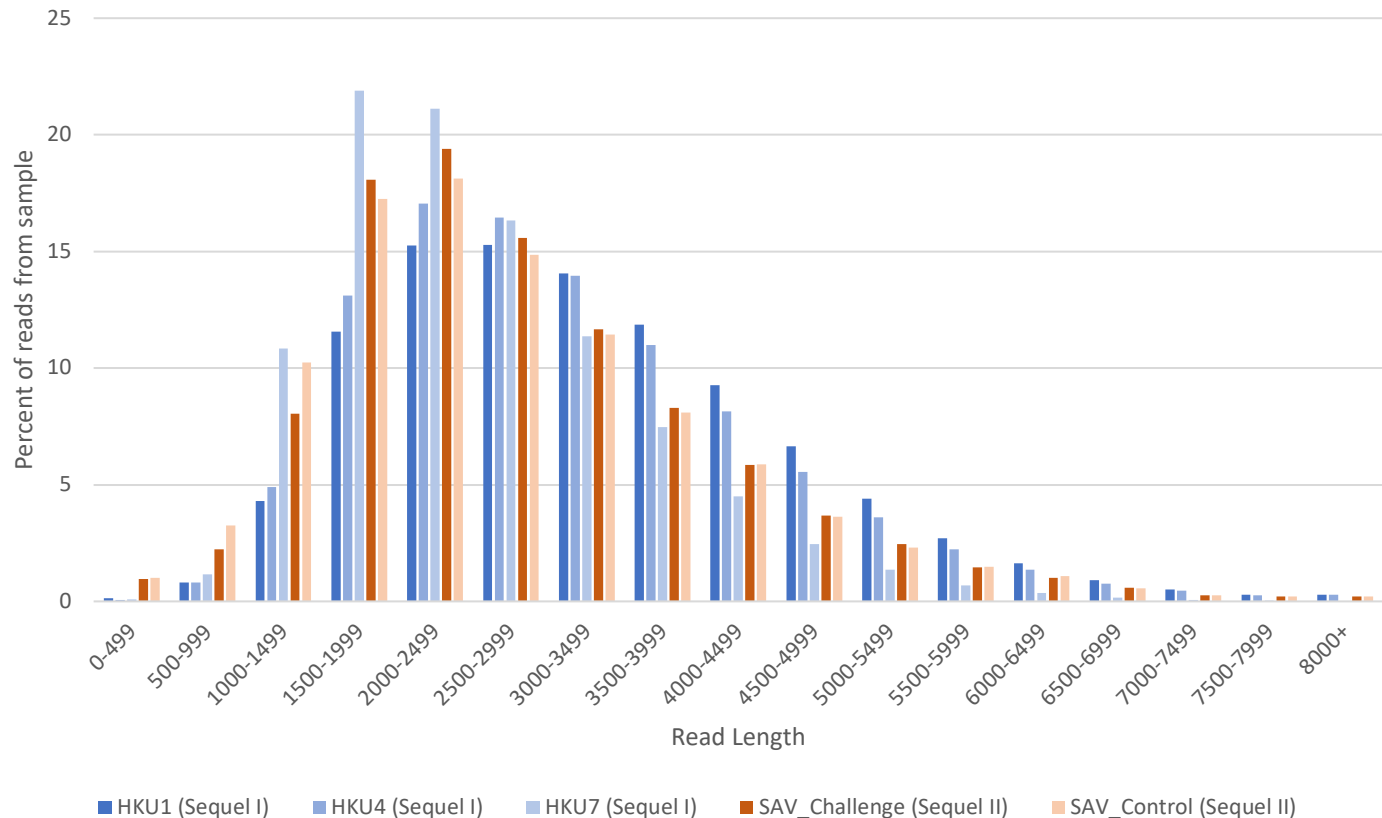

Supplement: Supplementary file 2 [file Data_Sheet_2.PDF]
